# Synthesis and high formaldehyde sensing properties of quasi two-dimensional mesoporous ZnSnO_3_ nanomaterials

**DOI:** 10.1039/c9ra01593k

**Published:** 2019-05-14

**Authors:** Bingshan Wang, Jinbao Yu, Xiaohong Li, Jun Yin, Meng Chen

**Affiliations:** Department of Bio-chemistry, Jingdezhen University Jingdezhen Jiangxi 333000 China wbs31@163.com; National Engineering Research Center for Domestic & Building Ceramics, Jingdezhen Ceramic Institute Jingdezhen Jiangxi 333000 China

## Abstract

Quasi two-dimensional (2D) mesoporous ZnSnO_3_ nanomaterials (QTMZNS) were synthesized by a simple template-free hydrothermal method. The as-prepared products were characterized by TEM, SEM, XRD, TG/DTA, and FTIR. The results showed that the precursor was a mixture of Zn_5_(OH)_6_(CO_3_)_2_ and ZnSnO_3_ in the hydrothermal process, and the high purity QTMZNS were obtained by calcination combined with subsequent washing of 20 wt% NH_4_Cl solutions. A possible growth process and mechanism of the quasi 2D mesoporous structure was proposed. Gas sensing properties of QTMZNS were investigated, and the QTMZNS-based sensors exhibited excellent gas sensing properties. When exposed to 100 ppm formaldehyde vapors, the response sensitivity is 45.8, and the concentration limit can reach as low as 0.2 ppm of formaldehyde. All these results are much better than those reported so far, which will have great potential applications for practical air quality monitoring.

## Abstract 2

Quasi two-dimensional mesoporous ZnSnO_3_ nanomaterials (QTMZNS) were synthesized and the QTMZNS-based sensor exhibited high sensitivity to formaldehyde vapors.

## Graphical abstract

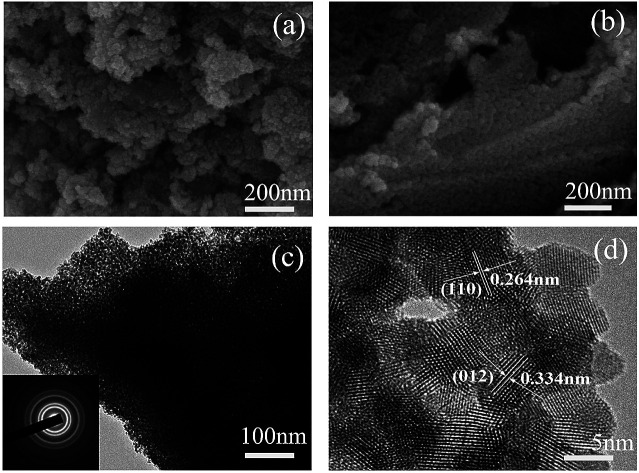

## 1. Introduction

Nanomaterials with porous structure have good potential applications in many fields, such as photocatalysis,^1^ super capacitors,^2^ drug delivery,^3^ lithium storage,^4^ gas sensing,^5,6^ and surface-enhanced Raman scattering (SERS).^7,8^ 2D sheet-like mesoporous materials with unique structural advantages have intriguing properties, attractive applications, as well as environmental and industrial benefits.^9–11^ Therefore, different kinds of mesoporous nanosheets materials were fabricated and have already achieved good success in many fields in recent years.^12–16^

In comparison to single metal oxides, composite metal oxides would have more superior properties than single metal oxides by altering the compositions.^17–19^ Therefore, the fabrication of composite metal oxides has attracted an increasing interest. ZnSnO_3_, as an important ternary semiconducting oxide, has been used widely in gas sensing fields, due to its high chemical response and excellent electronic properties.^20–27^ Because of its outstanding properties, ZnSnO_3_ has attracted extensive interest during the past few years, especially in terms of the synthesis of nanostructures. Until now, some ZnSnO_3_ porous structures such as porous ZnSnO_3_ hollow microspheres,^22^ porous ZnSnO_3_ hollow nanocubes,^23,24^ mesoporous ZnSnO_3_ cubes,^26^ and mesoporous ZnSnO_3_ nanocrystals^27^ have been successfully fabricated, whereas controlled synthesis of quasi two-dimensional mesoporous ZnSnO_3_ nanomaterials have not been reported. Lamellar nanosheet structure could endow the good stability of the material compared with nanoparticles due to the decreased surface energies, and stable nanosheet structure can ensure the stability of gas sensitive test results which can be seen in previous literatures.^28,29^ Porous structure is beneficial to gas diffusion and mass transport,^30,31^ the existence of defects such as oxygen vacancies could increase the chance of electrostatic interaction between testing gas and the surface of sensing materials, benefiting to oxygen adsorption and further enhance the gas response of the sensors.^32^ So, it is of significant value to develop porous ZnSnO_3_ nanosheets sensors.

In this paper, we adopted a general template-free hydrothermal method with subsequent calcination and washing of 20 wt% NH_4_Cl solutions for synthesis QTMZNS. The morphology, chemical composition, crystalline structure and thermal behavior of as-prepared products were characterized by SEM, TEM, XRD, and so on. Meanwhile, the formation mechanism has been investigated through the morphology evolution with different procedure. In addition, the formaldehyde sensing properties of the QTMZNS-based sensors were systematically studied. It is worth mentioning that the procedure for producing QTMZNS can easily be scaled-up, which is particularly attractive for industrial applications.

## 2. Experimental

### 2.1. Materials synthesis

All chemical reagents used in this experiment were of analytical grade and used without further purification. A typical synthesis procedure of the QTMZNS was as follows: 1.73 g of SnCl_4_·5H_2_O, 1.488 g of Zn(NO_3_)_2_, and 1.802 g of CO(NH_2_)_2_ were dissolved in 80 ml of deionized water together under magnetic stirring for 10 min at room temperature. Then the mixed solution was transferred to a Teflon-lined stainless steel autoclave and maintained at 130 °C for 16 h. After natural cooling, the precursor was filtered, washed with distilled water and alcohol for several times, and finally dried in air at 70 °C. The as-synthesized precursor was calcined at 500 °C for 3 h, and then washed by 20 wt% NH_4_Cl solutions for 10 min, filtered, washed with distilled water, and dried, thus the high purity QTMZN were obtained.

### 2.2. Characterization

The crystal phase, morphology and microstructure of the synthesized samples were characterized by X-ray diffraction (XRD: Bruker D8-Advance X-ray diffractometer, with high-intensity Cu K_α1_ radiation, *λ* = 1.5406 Å), Fourier transform infrared spectroscopy (FTIR, IR Prestige-21), field emission scanning electron microscopy (FE-SEM, Hitachi S-4800), and transmission electron microscopy (TEM; JSM-2010 HR, Japan). The pore diameter distribution and surface area of the products were tested by nitrogen adsorption/desorption analysis (Micromeritics ASAP 2020). Thermogravimetric and differential scanning calorimetry (TG-DSC) was performed on a NETZSCH STA449C thermogravimetric analyzer from 50 to 800 °C at a heating rate of 10 °C min^−1^ in N_2_ flow.

### 2.3. Fabrication and measurement of gas sensor

In order to study the gas sensing properties of the QTMZNS, gas sensors were obtained by coating the QTMZNS paste onto the surface of the ceramic tube with a pair of gold electrodes printed previously, and then a Cr–Ni alloy coil through the tube was used as a heater to form a side-heated gas sensor. Subsequently, the sensors were calcined at 460 °C for 3 h. Finally, in order to improve the long-term stability, the sensors were aged with 300 °C heating for 20 days. The gas-sensing properties of the QTMZNS were measured using a sensor tester of WS-30A (Weisheng Instruments Co., Ltd., China). The desired concentrations of the testing gases were obtained by the static liquid gas distribution method, which was calculated by the formula (*C* × 10^−6^ = *nRT*/0.018*P*. *P*: atmospheric pressure; *T*: room temperature; 0.018 m^3^: the test chamber volume). In the gas response measurement, specified testing liquid was injected into the closed 18L chamber through the injection pore and mixed with air. After the measurement, the sensor was exposed to the atmospheric air by opening the closed chamber to return to its initial state. The data were collected by a computer automatically. The gas response (*S*) was defined as the ratio of *R*_a_/*R*_g_ (*R*_a_: the resistance of the sensor in air; *R*_g_: the resistance of the sensor in testing gases). The recovery time and response time were defined as the time taken by the sensor to achieve 90% of the total resistance change during gas desorption and adsorption phase, respectively.

## 3. Results and discussion

### 3.1. Structure and morphology characterization

**Fig. 1:**
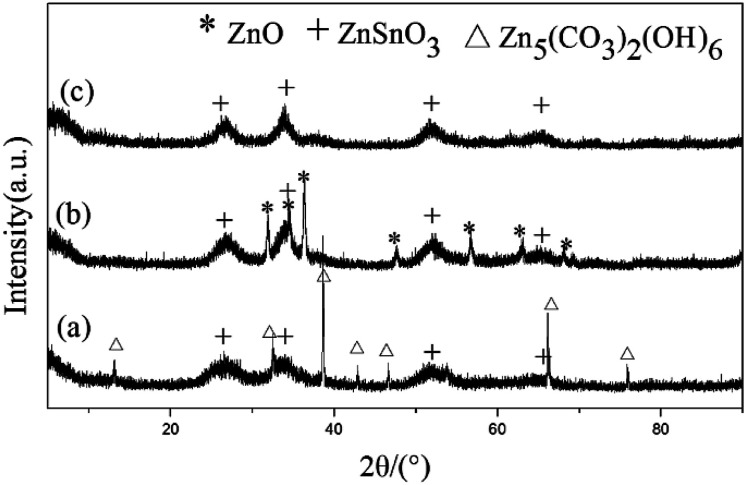
XRD patterns of the precursor (a) before annealing, (b) after annealing at 500 °C for 3 h, and (c) corresponding calcined product washed with 20 wt% NH_4_Cl solutions for 10 min. ZnSnO_3_, Zn_5_(CO_3_)_2_(OH)_6_, and ZnO peaks are marked with plus sign, triangular symbol, and asterisks respectively.

The high purity quasi two-dimensional mesoporous ZnSnO_3_ nanomaterials (QTMZNS) were obtained through a three-step procedure. First, the mixture precursor of Zn_5_(CO_3_)_2_(OH)_6_ and ZnSnO_3_ was derived from hydrothermal reaction process. Second, calcination of the mixture precursor could yield the mixed oxide of ZnO and ZnSnO_3_. Then the high purity sheet-like mesoporous ZnSnO_3_ nanomaterials were obtained by washing the corresponding calcined product with 20 wt% NH_4_Cl solutions for 10 min. Fig. 1 shows the XRD patterns of the precursor before annealing (Fig. 1(a)), after annealing at 500 °C for 3 h (Fig. 1(b)), and the final products which obtained by washing the corresponding calcined product with 20 wt% NH_4_Cl solutions for 10 min (Fig. 1(c)). From Fig. 1(a), the peaks in the XRD pattern can be well indexed to both the orthorhombic phase of ZnSnO_3_ (JCPDS no. 28-1486)^21^ and the monoclinic phase of Zn_5_(CO_3_)_2_(OH)_6_ (JCPDS no. 19-1458),^33^ which indicating that the precursor was the mixture of Zn_5_(CO_3_)_2_(OH)_6_ and ZnSnO_3_. For the precursor heat-treated at 500 °C for 3 h, the diffraction peaks including both ZnO (JCPDS no. 80-0074)^33^ and ZnSnO_3_ could be observed in Fig. 1(b), which was related to the mixed oxide of ZnO and ZnSnO_3_. As shown in Fig. 1(c), all diffraction peaks in the XRD pattern can be well-assigned to the standard pattern of ZnSnO_3_ (JCPDS no. 28-1486).^21^ No impurity phases are detected, which indicates that the final products are pure orth-ZnSnO_3_.

**Fig. 2:**
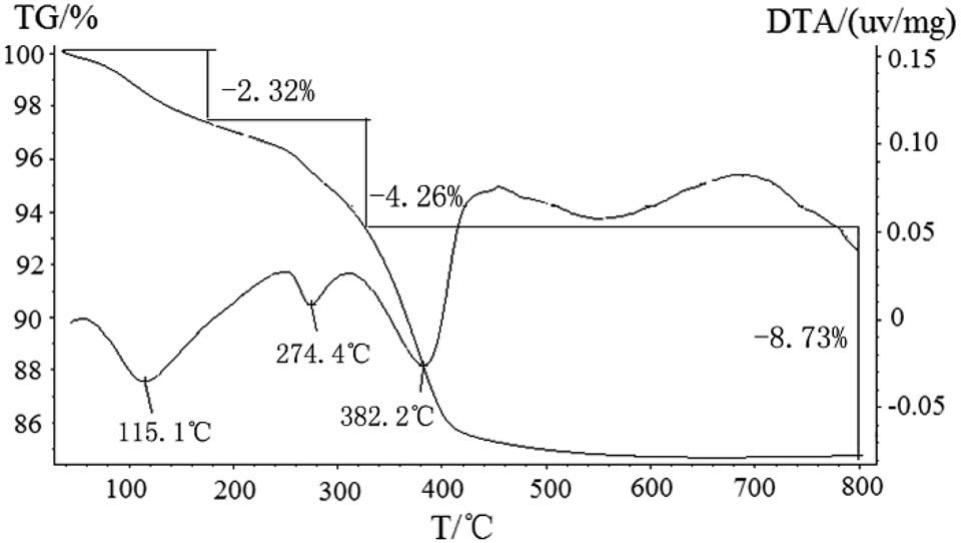
TG and DTA curves of the mixture precursor of (Zn_5_(CO_3_)_2_(OH)_6_ and ZnSnO_3_) at a heating rate of 10 °C min^−1^ in N_2_.

To trace the composition of the precursor and the formation history of ZnSnO_3_ during calcinations and washing with 20 wt% NH_4_Cl, TG/DTA and the FTIR spectra investigations were also carried out. Fig. 2 recorded the TG-DTA curve of the mixture precursor (Zn_5_(CO_3_)_2_(OH)_6_ and ZnSnO_3_), which carried out under N_2_ atmosphere, and the detected temperature range is from 50 °C to 800 °C at a heating rate of 10 °C min^−1^. The TG-DTA curves clearly show the mixture precursor has three weight loss steps in the detected temperature range. Obviously, the precursor before 150 °C has a weight loss, which is mainly attributed to residual moisture removing from the sample in pre-drying stage. After 150 °C, the TG-DTA curves show two weight loss in the temperature range of 150–500 °C with endothermic peaks at 274.4 °C and 382.2 °C, which can be attributed to the thermal decomposition of the precursor from Zn_5_(CO_3_)_2_(OH)_6_/ZnSnO_3_ to ZnO/ZnSnO_3_. The reaction equations are as follows:

1 Zn_5_(CO_3_)_2_(OH)_6_ → 3ZnO·2ZnCO_3_ + 3H_2_O (4.26 mass loss%)

2 3ZnO·2ZnCO_3_ → ZnO + 2CO_2_↑ (8.73 mass loss%)

**Fig. 3:**
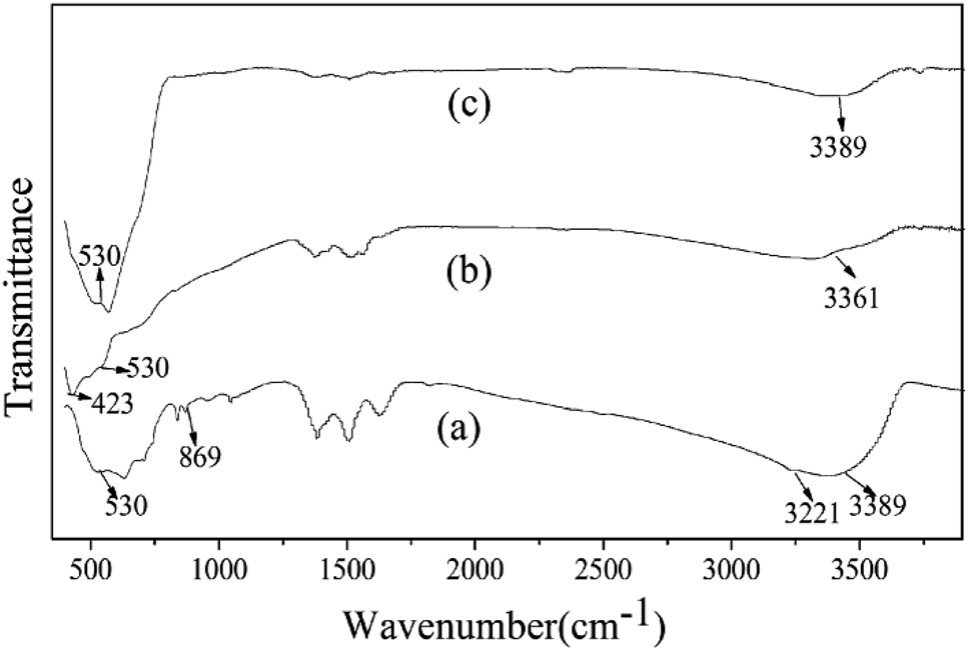
FTIR data of the precursors (a) before annealing, (b) after annealing at 500 °C for 3 h, and (c) corresponding calcined product washed with 20 wt% NH_4_Cl solutions for 10 min.

The value of total weight loss in this process was about 12.99%, which was not consistent with the theoretical value (25.96%). The result of the TG-TDA show there was other matter in the precursor, and which was consistent with the XRD results above. In the FTIR spectra shown in Fig. 3(a), the band at 3221 cm^−1^ was attributed to the bending vibration and stretching vibration modes of OH group in the structure of Zn_5_(CO_3_)_2_(OH)_6_. The band at 869 cm^−1^ was the stretching vibration of carbonyl group (C=O) for CO_3_^2−^.^34,35^ The band observed at 530 cm^−1^ was assigned to vibrations of Sn–O group for ZnSnO_3_.^21,46^ After calcination, the precursor at the peaks of the hydroxyl group and carbonyl group almost disappeared. And the band at 423 cm^−1^ which was assigned to stretching vibrations of Zn–O group for ZnO was shown in Fig. 3(b).^34,35^ However, the band at 423 cm^−1^ has disappeared in Fig. 3(c) when the corresponding calcined product washed with 20 wt% NH_4_Cl solutions for 10 min, indicating the loss of the ZnO, and then also proved that the final product is pure ZnSnO_3_, which is also consistent with the XRD results above. In addition, a much weaker broad peak at about 3380 cm^−1^ (Fig. 3(a)–(c)) is attributed to surface absorbed water after drying.

**Fig. 4:**
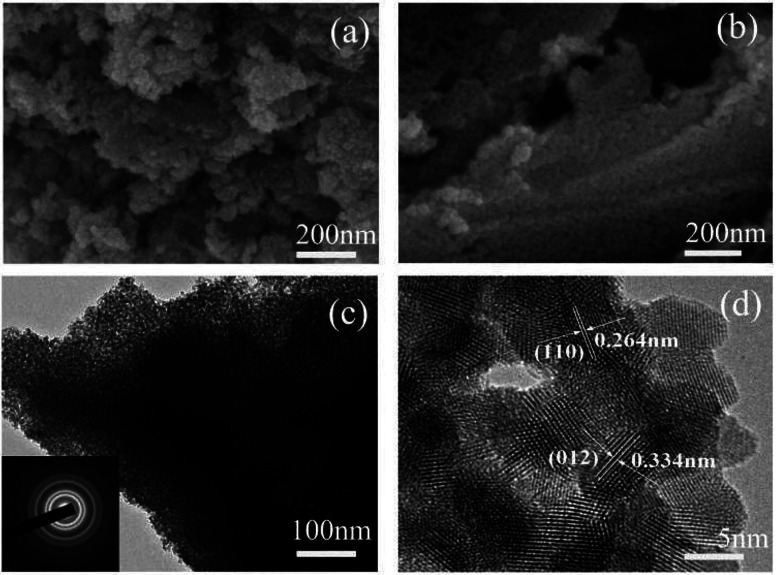
(a) and (b) SEM image, (c) TEM image, (d) HRTEM image of the pure ZnSnO_3_.

The morphology, structure and chemical composition of the as-formed ZnSnO_3_ were illuminated by SEM, TEM and HRTEM. The overall morphology of the sample, as shown in Fig. 4(a) and (b), indicates the irregular sheet-like structures which were randomly stacked together. The surface of the irregular sheet-like structures with lots of interspaces was composed of many tiny nanoparticles. Its typical TEM and HRTEM images, as shown in Fig. 4(c) and (d), further depict the sheet-like morphology and mesoporous structures of the pure ZnSnO_3_ which are uneven in thickness and uneven in size. The HRTEM image demonstrates that the quasi two-dimensional mesoporous ZnSnO_3_ nanomaterials (QTMZNS) were composed of many tiny nanoparticles with 5–10 nm size, and QTMZNS have nanopores in the main range 2–12 nm, which means that the QTMZNS could have larger active surfaces.^5,36^ Mainly, two sets of lattice spacings of 3.34 Å and 2.64 Å are observed, which correspond to (012) and (110) planes of orth-ZnSnO_3_, respectively. The inset in Fig. 4(c) shows the SAED pattern of the QTMZNS, indicating its polycrystalline nature, which further confirms the HRTEM's observation.

### 3.2. Growth process and mechanism

**Fig. 5:**
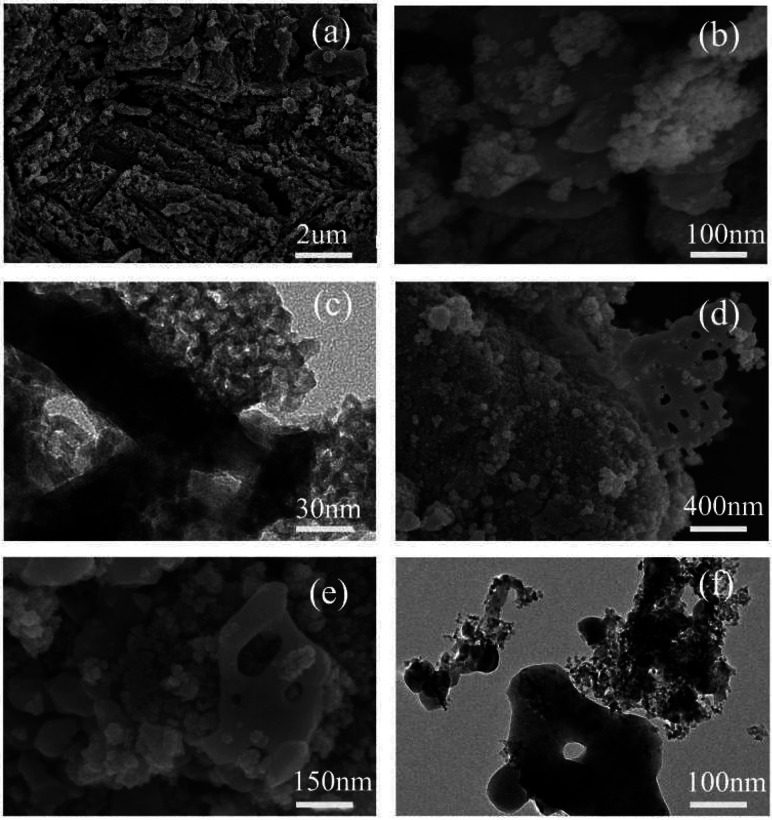
(a) Low, (b) high magnification SEM images and (c) TEM image of the mixture precursor. (d) Low, (e) high magnification SEM images and (f) TEM image of the mixture precursor after an annealing treatment at 500 °C.

To understand the formation process and the possible growth mechanism of the QTMZNS, the morphology, structure and chemical composition of the mixture precursor (Zn_5_(CO_3_)_2_(OH)_6_ and ZnSnO_3_) before and after calcination were also studied, as shown in Fig. 5. Fig. 5(a) is a typical low-magnification SEM image of the mixture precursor which obtained by a hydrothermal method at 130 °C for 16 h, from which some quasi sandwich structure or layer-like structure were observed. A high-magnification SEM image and TEM image are shown in Fig. 5(b) and (c), which further depict the quasi sandwich structure of the mixture precursor. The quasi-sandwich structure is composed of nanosheets and irregular mesoporous sheet-like nanomaterials, and most of them alternate in turn. Fig. 5(d)–(f) are SEM images and a TEM image of the mixture precursor after an annealing treatment at 500 °C for 3 h. By contrast, the morphology and structure of the mixture precursor has almost no change before and after the calcination, except for the porous in the nanosheets. According to the above analysis results, including the Fig. 4 above, it is not difficult to find that the nanosheets morphology belongs to Zn_5_(CO_3_)_2_(OH)_6_ in Fig. 5(a)–(c), the porous nanosheets are ZnO in Fig. 5(d)–(f), and the quasi 2D mesoporous materials are ZnSnO_3_ in Fig. 4 and 5.

**Fig. 6:**
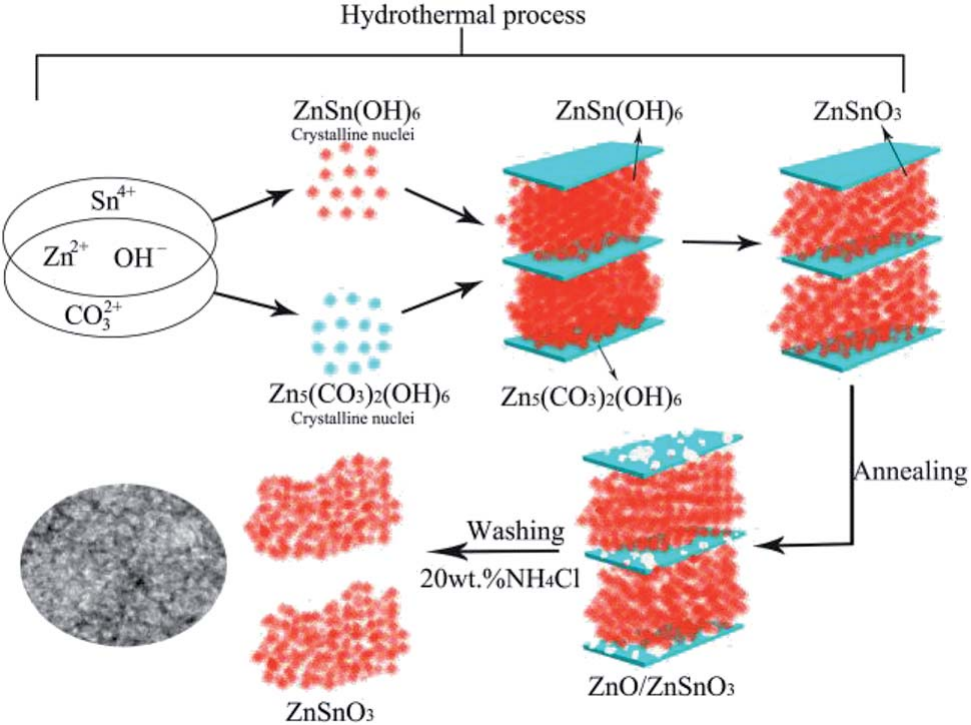
Schematic illustration for the possible formation process of QTMZNS.

On the basis of the above analysis results, a formation mechanism of the QTMZNS could be proposed here. A possible growth process was schematically illustrated in Fig. 6 though the exact mechanism was still unclear at present. The synthesis consists of three steps—hydrothermal process, annealing process and washing process with 20 wt% NH_4_Cl solutions. These processes may be explained by the following sequence of reactions:

3 CO(NH_2_)_2_ + H_2_O → 2NH_3_↑ + CO_2_↑

4 NH_3_ + H_2_O → NH_4_^+^ + OH^−^

5 CO_2_ + 2OH^−^ → CO_3_^2−^ + H_2_O

6 5Zn^2+^ + 2CO_3_^2−^ + 6OH^−^ → Zn_5_(CO_3_)_2_(OH)_6_↓

7 Zn^2+^ + Sn^4+^ + OH^−^ → ZnSn(OH)_6_↓ → ZnSnO_3_↓ + H_2_O

8 Zn_5_(CO_3_)_2_(OH)_6_ → 5ZnO + 2CO_2_↑ + 3H_2_O

9 NH_4_^+^ + H_2_O → H^+^ + NH_4_OH

10 2H^+^ + ZnO → Zn^2+^ + H_2_O

In the hydrothermal process, urea could produce OH^−^ anions and CO_3_^2−^ anions, which would react with the Zn^2+^ cations to form insoluble Zn_5_(OH)_6_(CO_3_)_2_. Numerous studies have shown that the crystal structure of the metallic basic salt with the molecular composition of M_*a*_(OH)_*b*_(X^*c*−^)_(2*a*−*b*)/*c*_ (M = Zn^2+^, Co^2+^, Ni^2+^, and so on) is sheets,^37–39^ and researchers have prepared a variety of two-dimensional porous ZnO nanomaterials using this principle in recent years.^40–43^ At the same time, OH^−^ anions and Zn^2+^ cations would also react with the Sn^2+^ cations to form ZnSn(OH)_6_ between sheets of Zn_5_(OH)_6_(CO_3_)_2_, which decomposed into orth-ZnSnO_3_ under the hydrothermal condition, and the quasi 2D mesoporous orth-ZnSnO_3_ nanomaterials are formed immediately. The reactions can be expressed by eqn (3)–(7). In the annealing process, the Zn_5_(OH)_6_(CO_3_)_2_ could be decomposed into wurtzite ZnO after further annealing at 500 °C for 3 h. Due to the loss of volatile gas such as H_2_O and CO_2_ released during the annealed process, the ZnO nanosheets with porous structure were formed, which could be expressed by eqn (8). In the washing process with 20 wt% NH_4_Cl solutions, the hydrolysis of NH_4_Cl produced a suitable acidic environment that was just capable of reacting with ZnO rather than ZnSnO_3_, which could be expressed by eqn (9) and (10). So after the three synthesis steps, the final products were pure quasi 2D mesoporous orth-ZnSnO_3_ nanomaterials. In our synthesis process, neither the soft template nor the hard template was used, but the nanosheets Zn_5_(OH)_6_(CO_3_)_2_ formed in the hydrothermal process played the role of template. This method was worthy of reference and recommendation.

### 3.3. Gas sensing properties of QTMZNS

**Fig. 7:**
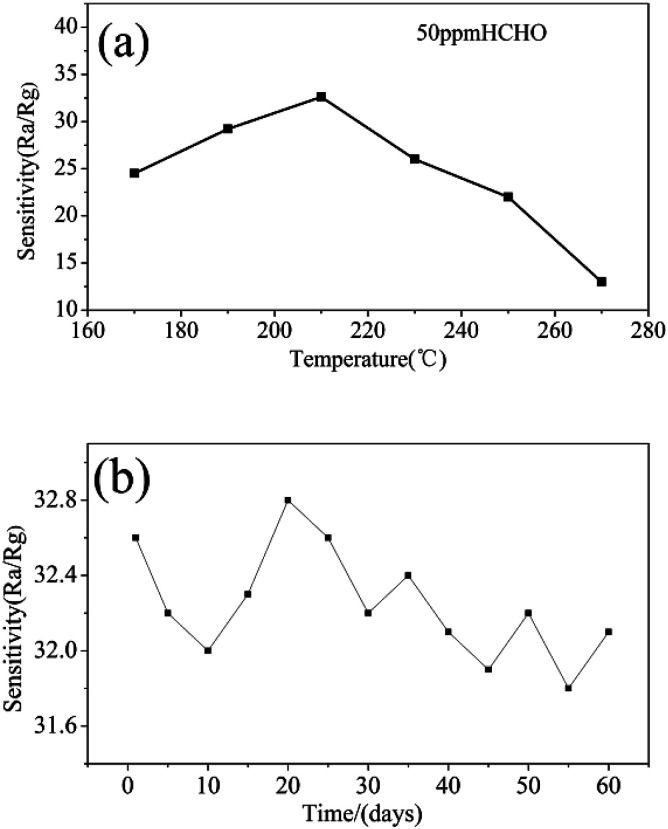
(a) Response *versus* working temperature of the QTMZNS-based sensor exposed to 50 ppm HCHO, (b) long-term stability of the QTMZNS-based sensor exposed to 50 ppm HCHO at 210 °C.

**Fig. 8:**
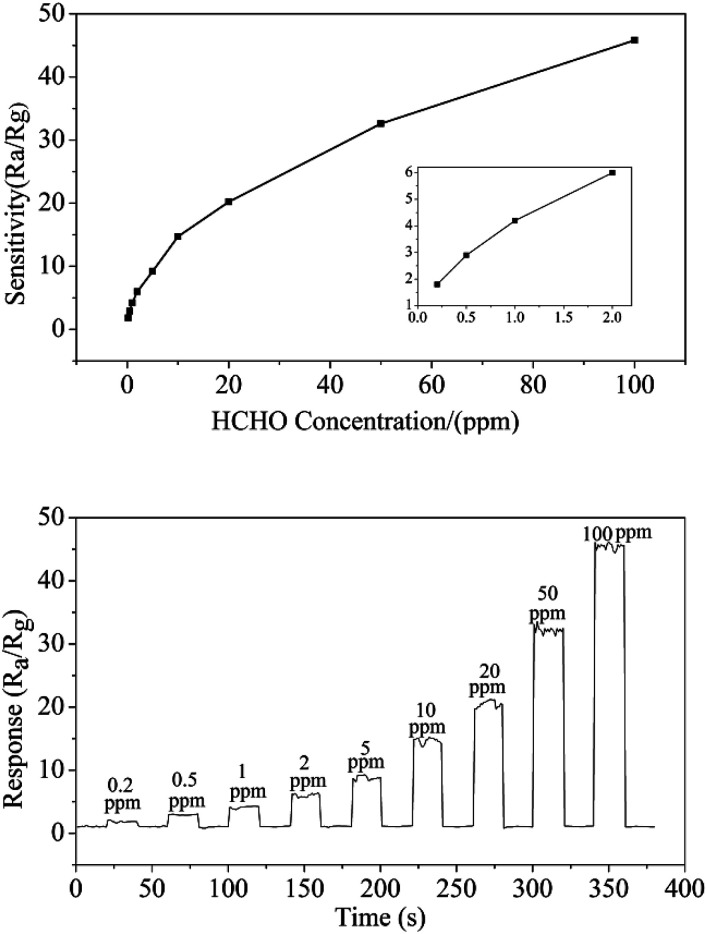
The response of QTMZNS to different concentration of formaldehyde gas at the working temperature of 210 °C.

To the semiconductor oxide sensors, gas sensing properties depend strongly on the working temperature. Fig. 7(a) shows response *versus* working temperature of the QTMZNS-based sensors exposed to 50 ppm HCHO. It can be seen that the response to formaldehyde increased with the increase of working temperature from 170 to 210 °C, and reached maximum response value at 210 °C. After that, the response value decreased with increasing temperature. Therefore, the optimum working temperature of the sensors based on QTMZNS could be selected as 210 °C. The long-term stability of a gas sensor is necessary for practical applications. Good stability or good reproducibility needs the reliability guarantee of the material. The results in Fig. 7(b) showed that the QTMZNS-based sensor tended to remain relatively stable during a long-term stability measurement of 60 days. The QTMZNS-based sensor showed a decrease of about 1.5%, implying a good reliability, which also indicated that the QTMZNS-based sensors obtained good repeatability for HCHO detection. Fig. 8 displays the response of QTMZNS to different concentration of formaldehyde gas at the working temperature of 210 °C. It can be seen that the QTMZNS-based sensor provided a stable baseline in the air condition. After HCHO was injected, the QTMZNS-based sensor got a positive response. When the QTMZNS-based sensor was exposed in air again, it presented a recovery characteristic. With the corresponding formaldehyde gas concentration increasing, the response of the QTMZNS-based sensor increased. The response of the sensor based on QTMZNS to 0.2, 0.5, 1, 2, 5, 10, 20, 50, and 100 ppm were 1.8, 2.9, 4.2, 6.0, 9.2, 14.7, 20.2, 32.6, and 45.8, respectively.

**Fig. 9:**
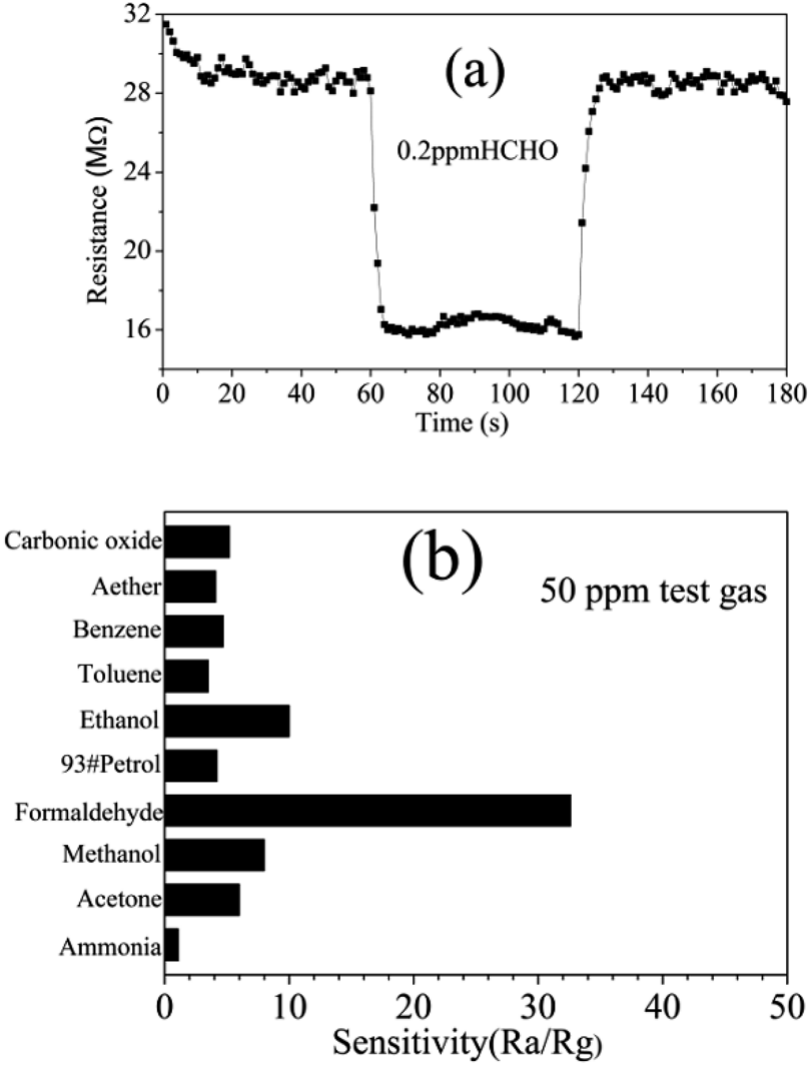
(a) Response/recovery time graph of the QTMZNS-based sensor to 0.2 ppm formaldehyde at 210 °C. (b) Selectivity of the QTMZNS-based sensor to 50 ppm different gases at 210 °C.

The response/recovery time is an important parameter for the application of gas sensors.^44^Fig. 9(a) shows the response/recovery time graph of the QTMZNS-based sensor to 0.2 ppm formaldehyde at 210 °C. It can be observed that the QTMZNS-based sensor exhibits a short response–recovery time and low detection limit for the detection of formaldehyde. The response and recovery times were about 3 s and 6 s when the formaldehyde concentration was 0.2 ppm. For the semiconductor sensors, good selectivity is also a critical parameter and it is the ability of sensors to respond to a specific gas in presence of other gases.^45^ The target gases, such as ammonia, ethanol, acetone, formaldehyde, carbonic oxide, aether, benzene, 93#petrol, methanol, and toluene were used to investigate the sensor selectivity at an optimal working temperature of 210 °C, where the concentration of all the testing gases was 50 ppm. It is can be seen clearly from Fig. 9(b), the sensor based on QTMZNS has a much higher response to formaldehyde than that to ethanol, methanol, acetone, carbonic oxide, benzene, 93#petrol, toluene, and ammonia under fixed concentration. So the result indicates that the sensor based on the QTMZNS exhibited low detection limit, high sensitivity, great selectivity and a short response–recovery time for detection of formaldehyde vapors at a low operating temperature of 210 °C, which will have great potential in applications for practical air quality monitoring. To further consider the application value of the QTMZNS-based sensor, a comparison of the formaldehyde sensing ability of different ZnSnO_3_ materials between our work and previous literatures is listed in Table 1.^24–26,46–49^ The response (45.8) of the QTMZNS to formaldehyde is higher than that of other ZnSnO_3_ materials. Moreover, the present sensor can detect HCHO sensing properties at 0.2 ppm, and such a low-concentration formaldehyde gas test has not been reported so far to ZnSnO_3_ sensors.

**Table 1:** Comparison of formaldehyde sensing ability of different gas sensors based on ZnSnO_3_ materials

Materials	HCHO concentration (ppm)	Working temperature (°C)	Response (*R*_g_/*R*_a_)	References
ZnSnO_3_ porous cubes	100	300	36.8	46
ZnSnO_3_ nanocages	50	210	∼5	47 and 48
ZnSnO_3_ cubic crystallites (200 nm)	100	400	∼15	49
ZnSnO_3_ solid cubes	100	220	10.7	24
ZnSnO_3_ single-shelled cubes	100	220	15.4	24
ZnSnO_3_ multishelled cubes	100	220	37.2	24
ZnSnO_3_ hollow spheres	50	270	<5	25
ZnO/ZnSnO_3_ mixed oxides	50	370	4.1	50
ZnSnO_3_ quasi 2D nanomaterials	100	210	45.8	This work

### 3.4. Sensing mechanism of QTMZNS

**Fig. 10:**
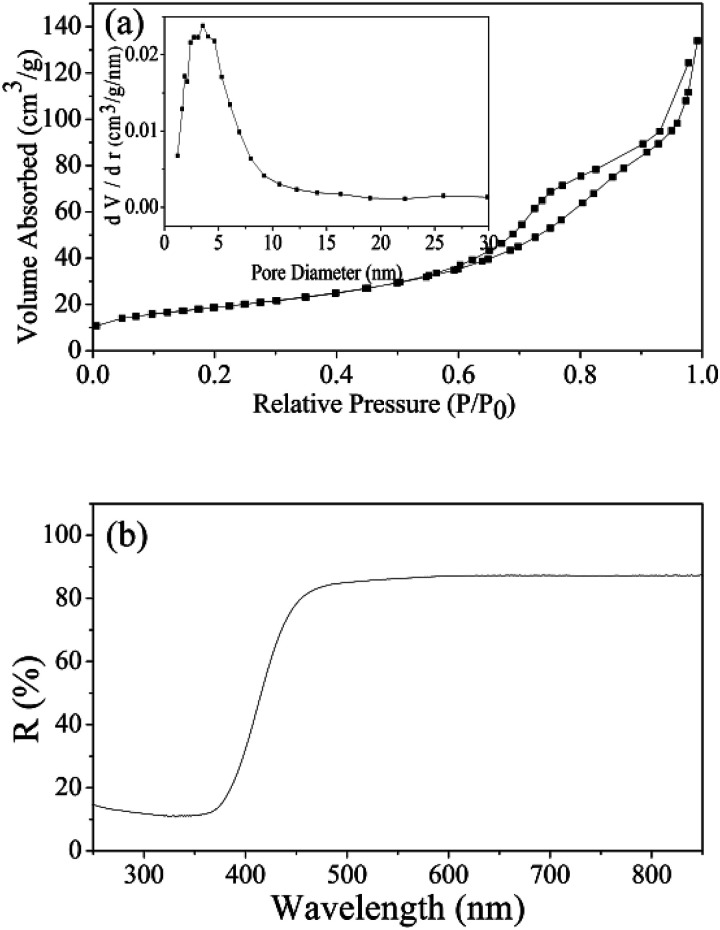
(a) Nitrogen adsorption–desorption isotherms and pore size distribution curves (the inset), and (b) UV-visible DRS spectra of QTMZN.

For n-type ZnSnO_3_ material, the sensing mechanism that is widely accepted is the electrical conductivity taken place in the surface of the materials, which involves serial processes of adsorption–oxidation–desorption.^50,51^ When QTMZNS are in the air, mass of oxygen molecules are adsorbed on the surface, and electrons from the surface of QTMZNS sensing layer are seized by lots of oxygen adsorbed, which can give rise to the transformation from oxygen species to oxygen ions (O^−^) at the operating temperature and the formation of electron depletion layers. As a result, the potential barriers are formed and the resistance is relatively high. After exposed to formaldehyde gas, the interaction occurs between formaldehyde gas and surface oxygen species and the electrons are released back into the sensing material, which results in a dramatic decrease in resistance. Therefore, an increase in surface area, chemisorbed oxygen and open nanoholes can improve the sensing performance. In this work, in order to better explain the outstanding gas-sensing properties for formaldehyde vapors at a relative low operating temperature of the QTMZNS-based sensor, both the BET specific surface area and a band gap of the QTMZNS were also investigated, and the result shows in Fig. 10. Nitrogen adsorption–desorption analysis of the QTMZNS (Fig. 10(a) and inset) reveals that the specific surface area using BET method is about 105.3 m^2^ g^−1^ and the average pore size of such a sample is about 7.9 nm, which is corresponding with the SEM and TEM in Fig. 4. The specific surface area of the QTMZNS is bigger than that of other ZnSnO_3_ material, such as ZnSnO_3_ solid cubes (37 m^2^ g^−1^),^20^ ZnSnO_3_ nanosheets (57.86 m^2^ g^−1^),^21^ ZnSnO_3_ hollow microspheres (30.21 m^2^ g^−1^),^22^ porous ZnSnO_3_ hollow nanocube (66.9 m^2^ g^−1^),^23^ ZnSnO_3_ single-shelled cubes (70 m^2^ g^−1^), ZnSnO_3_ double-shelled cubes (86 m^2^ g^−1^), ZnSnO_3_ multishelled cubes (98 m^2^ g^−1^),^24^ mesoporous ZnSnO_3_ nanocrystals (96 m^2^ g^−1^),^27^ and so on. This isotherm can be classified as type IV with an H3 type hysteresis loop which is characteristic for mesoporous materials.^52^Fig. 10(b) represents the UV-visible diffused reflectance spectra of the QTMZNS. The UV-visible diffused reflectance spectrum of QTMZNS shows absorption edge at 413 nm giving a band gap (*E*_g_ = *hc*/*λ*) value of 3.0 eV, which is the lowest than those of the previous reports about ZnSnO_3_ material.^48,53^ The decrease of band gap energy can be attributed to surface defects, and the defects might be the vacancies of oxygen/defects. As a result, the surface-related defects are the best condition to the absorption of O^2−^ and O^−^ for gas detection.^48,54,55^ According to the HCHO sensing mechanism of ZnSnO_3_ above, the outstanding gas-sensing properties for formaldehyde vapors of the QTMZNS-based sensor were attributed to the unique structure of the QTMZNS which have the following four advantages: (a) large specific surface area provides large contact area and void which can offer more chemical active sites. (b) Less band gap energy should help the O_2_ adsorption on the ZnSnO_3_ surface to trap electrons from the conduction band of ZnSnO_3_ and enhance the sensing performance. (c) Special open nanoholes can facilitate the adsorption of target gas and shorten gas diffusion path. (d) QTMZNS have been an aggregate assembled by nanoparticles, which endow the good stability compared with particles due to the decreased surface energies. Stable structure can ensure the stability of gas sensitive test results which can be seen in previous literatures.^28,29^ So, choosing QTMZNS as a sensitive material is a good choice for the detecting of HCHO.

## 4. Conclusions

In summary, the quasi two-dimensional mesoporous ZnSnO_3_ nanomaterials were synthesized by a simple template-free hydrothermal method combined with calcination and subsequent washing. The appearance of the quasi two-dimensional mesoporous structure was attributed to the decomposition of ZnSn(OH)_6_ and the formation of nanosheets Zn_5_(OH)_6_(CO_3_)_2_ in the hydrothermal process. The QTMZNS-based sensors exhibited outstanding performances towards formaldehyde, which are suitable for continuous and effective detection of formaldehyde. It is anticipated that the unique sensing properties of QTMZNS make them a good potential material in the gas sensor field.

## Conflicts of interest

There are no conflicts to declare.

## Supplementary Material
